# Advances in γδ T cell-mediated immune surveillance in cervical cancer

**DOI:** 10.3389/fonc.2026.1919105

**Published:** 2026-09-28

**Authors:** Jinlei Wang, Xiujun Xu, Qiang Li

**Affiliations:** Department of Gynaecology, Shengli Oilfield Central Hospital, Shandong, China

**Keywords:** adoptive immunotherapy, cervical cancer, HPV, immune surveillance, immunotherapy, γδ T cells

## Abstract

Cervical cancer is one of the most common malignancies of the female reproductive system worldwide, and its development is closely associated with persistent infection with high-risk human papillomavirus (HPV). γδ T cells, as key effector cells bridging innate and adaptive immunity, exert unique and complex regulatory functions in tumor immune surveillance. However, the distribution patterns, functional polarization states, and antitumor mechanisms of distinct γδ T-cell subsets within the cervical cancer microenvironment have not yet been systematically elucidated, and the impact of HPV infection on the functional remodeling of γδ T-cell subsets remains controversial. This review summarizes the distribution characteristics and functional polarization states of the major γδ T-cell subsets, including Vδ1, Vδ2, and Vδ3 T cells, in the cervical cancer microenvironment. We further discuss the molecular mechanisms through which these subsets participate in immune surveillance via T-cell receptor (TCR) signaling, NKG2D-mediated recognition, Fas/FasL signaling, and cytokine secretion, and examine the regulatory effects of HPV infection on γδ T-cell subset remodeling. In addition, recent preclinical advances in γδ T-cell subset-based adoptive immunotherapy and combination strategies involving immune checkpoint blockade in cervical cancer are reviewed. Overall, this review aims to provide a theoretical basis and new perspectives for optimizing immunotherapeutic targets and developing individualized treatment strategies for cervical cancer.

## 1 Introduction

Cervical cancer remains a major cause of cancer-related mortality among women and is closely associated with persistent infection with high-risk human papillomavirus (HPV), particularly HPV16 and HPV18 (1). Although HPV vaccination and screening have reduced disease incidence, outcomes for advanced and recurrent cervical cancer remain poor, and conventional treatments provide limited benefit (2). Rare aggressive subtypes, such as small-cell neuroendocrine carcinoma, also respond poorly to standard chemotherapy and immune checkpoint inhibitor monotherapy (3). γδ T cells are unconventional lymphocytes with features of both innate and adaptive immunity and can recognize tumor cells in an MHC-independent manner through T-cell receptors (TCRs) and activating receptors such as NKG2D. However, their roles in cervical cancer are complex because γδ T-cell infiltration and function may vary according to subset composition and the tumor genomic landscape (4).

Persistent HPV infection may further reshape γδ T-cell immunity through multiple immune-evasion mechanisms. HPV16 E6 promotes p53 degradation (5), whereas E7 disrupts pRb and interferon-related signaling (6), potentially altering both innate and adaptive antitumor responses. Although direct evidence linking HPV oncoproteins to γδ T-cell dysfunction remains limited, HPV-associated changes in cytokines such as IL-12 and IL-18 may indirectly regulate γδ T-cell activation. Recent single-cell and spatial transcriptomic technologies now enable high-resolution characterization of γδ T-cell heterogeneity, functional states, and spatial interactions with tumor cells, CD8^+^ T cells, and dendritic cells (7). These advances may help clarify the distinct roles of Vδ1, Vδ2, and Vδ3 subsets and facilitate the development of γδ T-cell-based strategies, including adoptive cell transfer, bispecific antibodies, and CAR-engineered γδ T cells.

## 2 Classification and functional characteristics of γδ T cell subsets

γδ T cells are unconventional T lymphocytes that develop in the thymus through V(D)J recombination and can be broadly classified into Vδ1, Vδ2, and Vδ3 subsets according to usage of the TCR δ-chain variable region (8). These subsets display distinct tissue distributions, phenotypic characteristics, and functional properties. Vδ1 T cells are preferentially enriched in epithelial and mucosal tissues, including the intestine, skin, and female reproductive tract, and constitute an important tissue-resident γδ T-cell population in the cervical mucosa, where they frequently express residency-associated markers such as CD103 and CD69 (9). In contrast, Vδ2 T cells, predominantly represented by the Vγ9Vδ2 subset, account for approximately 50%–90% of circulating γδ T cells and are characteristically activated by phosphoantigens such as isopentenyl pyrophosphate (IPP) and HMB-PP. Their abundance in cervical mucosal tissues is relatively low under physiological conditions but may increase following inflammatory or tumor-associated recruitment (10). Vδ3 T cells are much less abundant under homeostatic conditions but may expand during chronic viral infection and in selected tumor microenvironments, although their biological significance remains insufficiently characterized (1). The differentiation and functional polarization of γδ T-cell subsets are further shaped by transcription factors such as RORγt, Eomes, and T-bet, together with cytokines including IL-7 and TGF-β, thereby influencing their tissue-homing and effector programs (11). In cervical cancer, γδ T-cell abundance also exhibits substantial interpatient heterogeneity, which may partly reflect differences in the somatic mutational landscape and may have potential prognostic significance (8).

The distinct antitumor functions of γδ T-cell subsets are closely linked to their different antigen-recognition and activation mechanisms. Vδ1 T cells can respond to stress-associated molecules such as MICA, MICB, and ULBP family members expressed by transformed or HPV-infected epithelial cells, and their activation can be further enhanced through receptors such as NKG2D (12). By contrast, Vγ9Vδ2 T cells sense abnormalities in intracellular phosphoantigen metabolism through a butyrophilin-dependent mechanism involving BTN3A1 and BTN2A1 (13). Increased activity of the mevalonate pathway in tumor cells can lead to IPP accumulation, which is sensed through BTN3A1 and communicated to the Vγ9Vδ2 TCR in cooperation with BTN2A1, resulting in rapid activation (10). The recognition biology of Vδ3 T cells remains less well defined, although emerging evidence suggests that some Vδ3 T cells may recognize CD1d-presented lipid antigens (14). In addition to TCR signaling, γδ T-cell activity is regulated by activating receptors such as NKG2D and DNAM-1 and inhibitory receptors including KIRs and PD-1, indicating that their functional output is determined by the balance between stimulatory and suppressive signals within the tumor microenvironment (15). Notably, persistent BTN3A1-associated signaling in cervical cancer has also been linked to induction of NR4A2/NR4A3 and an exhaustion-like phenotype in Vδ2 T cells, highlighting the context-dependent consequences of sustained γδ T-cell activation (13).

## 3 Distribution and phenotypic characteristics of γδ T-cell subsets in the cervical cancer microenvironment

### 3.1 Alterations in γδ T-cell subset proportions in tumor tissues and peripheral blood and their clinical relevance

Multiple immunohistochemical and flow cytometric studies have characterized γδ T-cell infiltration in cervical cancer tissues. γδ T cells have been reported to account for approximately 5%–15% of total CD3^+^ T cells within cervical cancer tissues, a proportion higher than that observed in normal cervical tissues (16, 17). However, substantial variability in infiltration density has been reported across studies, highlighting considerable interpatient heterogeneity (14). This heterogeneity may be associated, at least in part, with the somatic mutational landscape of individual tumors, as differences in tumor mutational burden and mutation profiles may influence the recruitment, activation, and expansion of γδ T cells. Distinct γδ T-cell subsets display markedly different distribution patterns within the cervical cancer microenvironment. Vδ1 T cells represent the predominant tumor-infiltrating subset and may constitute approximately 60%–80% of infiltrating γδ T cells, whereas the proportion of circulating Vδ2 T cells is significantly reduced (14). This altered distribution may reflect selective recruitment of Vδ2 T cells into tumor tissues or, alternatively, increased apoptosis or functional attrition of these cells within the tumor microenvironment. Further studies have suggested that an increased intratumoral Vδ1/Vδ2 ratio is positively associated with advanced FIGO stage, lymph-node metastasis, and lymphovascular invasion. However, its relationship with overall survival has been inconsistent across studies, suggesting that the functional state of γδ T-cell subsets, rather than their absolute abundance alone, may have greater prognostic significance (8, 14). Recent single-cell sequencing studies have further revealed substantial heterogeneity among tumor-infiltrating Vδ1 T cells in cervical cancer. These cells can be subdivided into distinct functional states, including a cytotoxic Vδ1 effector population characterized by high expression of GZMB, PRF1, and GNLY, as well as an immunoregulatory Vδ1 population expressing relatively high levels of IL-10, TGF-β, and CTLA-4 (1, 18). By contrast, Vδ3 T cells remain rare within cervical cancer tissues. Nevertheless, expansion of Vδ3 T cells has been reported in a subset of HPV16-positive patients and appears to be associated with increased expression of inhibitory receptors such as PD-1 and TIM-3 (19). The clinical significance of this rare population remains unclear and requires validation in larger patient cohorts and dedicated functional studies.

### 3.2 Effects of HPV infection on the composition and function of γδ T-cell subsets

HPV infection is an important factor shaping the composition and functional state of γδ T-cell subsets within the cervical cancer microenvironment. HPV E6 and E7 oncoproteins may increase the expression of stress-associated ligands such as MICA and MICB on cervical epithelial cells, thereby promoting the early activation of γδ T cells. At the same time, HPV can interfere with components of the antigen-processing machinery, including TAP1 and tapasin, potentially impairing the stable presentation of activating ligands and contributing to immune evasion (20). In addition, HPV16 E5 can downregulate MHC class I expression and interfere with the membrane localization of NKG2D ligands, thereby reducing the efficiency of immune recognition while simultaneously remodeling the local immune microenvironment through EGFR-dependent signaling (21). Persistent HPV infection may also contribute to progressive γδ T-cell dysfunction. Chronic antigenic stimulation has been associated with an exhausted phenotype in Vδ2 T cells, characterized by increased PD-1 expression, reduced CD107a mobilization, and impaired production of IFN-γ and TNF-α (22, 23). In contrast, under conditions of prolonged stimulation, Vδ1 T cells may preferentially acquire an IL-17-producing phenotype, which could contribute to inflammatory tumor progression (24). The cytokine milieu generated during persistent HPV infection further contributes to γδ T-cell remodeling. Increased levels of TGF-β and IL-10 within the cervical microenvironment can activate SMAD- and STAT3-dependent signaling pathways, respectively, thereby promoting the acquisition of immunoregulatory phenotypes and suppressing the overall antitumor activity of γδ T cells (25). These findings suggest that HPV infection exerts complex and stage-dependent effects on γδ T-cell subsets. The ultimate functional outcome is likely determined by the duration of infection, the expression patterns of viral oncoproteins, and the combined influence of local cytokine, metabolic, and checkpoint signals (**Figure 1**).

**Figure 1:**
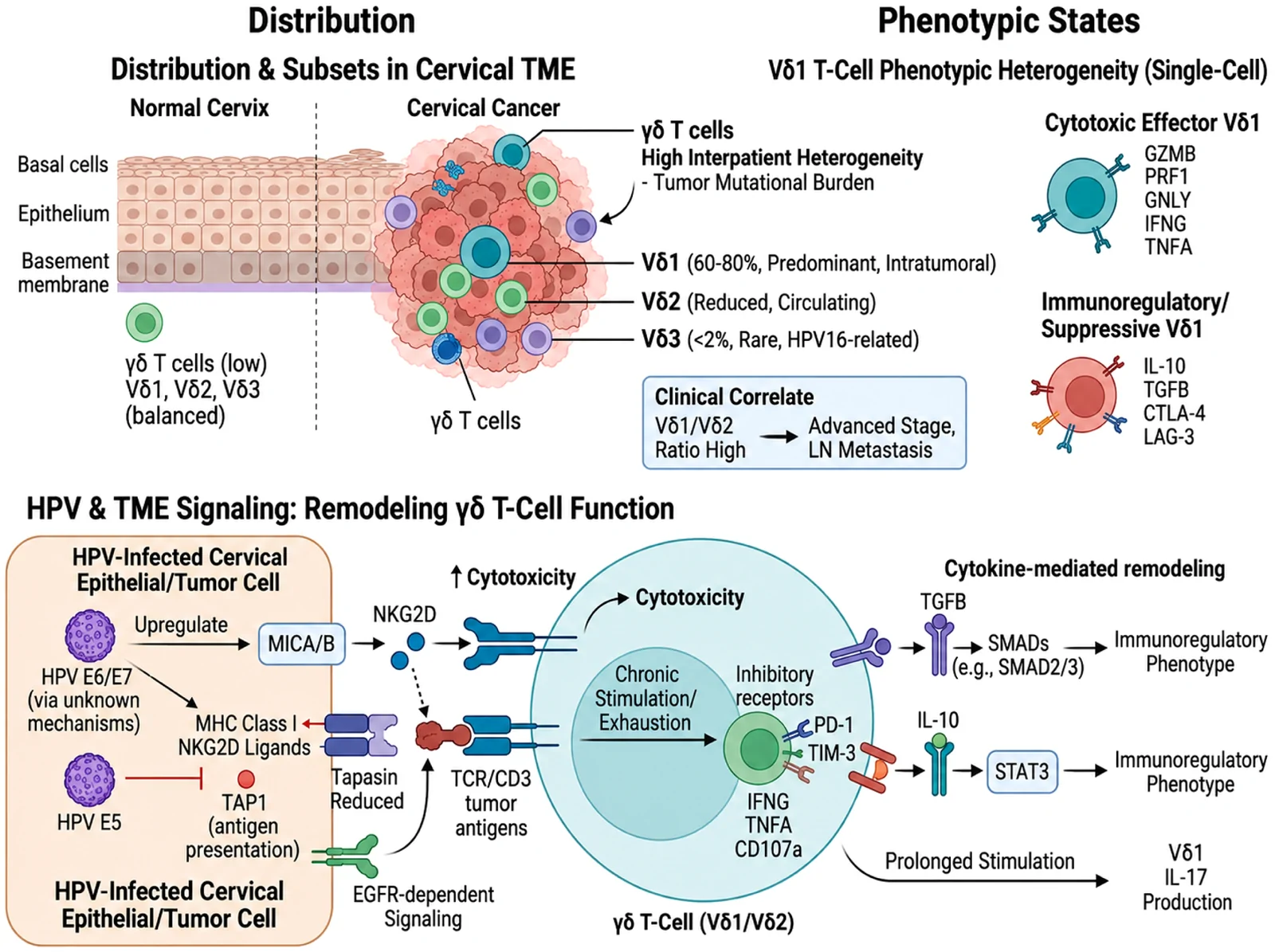
Distribution and phenotypic characteristics of γδ T-cell subsets in the cervical cancer microenvironment.

## 4 Effector mechanisms of γδ T-cell subsets in immune surveillance of cervical cancer

### 4.1 Direct and indirect cytotoxic mechanisms

γδ T cells exert antitumor cytotoxicity against cervical cancer through multiple direct and indirect mechanisms. First, activated Vδ1 T cells can recognize cervical cancer cells through coordinated signaling via the T-cell receptor (TCR), followed by the release of cytotoxic granules containing perforin and granzymes (26, 27). Perforin forms pores in the target-cell membrane, allowing granzymes to enter the cytoplasm and activate caspase-dependent apoptotic pathways, ultimately leading to tumor-cell death (28). Second, activated γδ T cells express high levels of Fas ligand (FasL), which binds to Fas on cervical cancer cells and triggers the extrinsic apoptotic pathway. This mechanism may be particularly effective against Fas-positive cervical cancer cells, whereas Fas-negative tumor cells are more dependent on perforin/granzyme-mediated killing (29). In addition, γδ T cells can induce apoptosis through the TNF-related apoptosis-inducing ligand (TRAIL)/TRAIL receptor axis. TRAIL receptors are frequently expressed at higher levels on cervical cancer cells than on normal cervical epithelial cells, providing a potential basis for preferential tumor-cell killing (30). Beyond direct cytotoxicity, γδ T cells can also enhance the activity of other immune effector populations. IFN-γ secreted by activated γδ T cells can upregulate MHC class I molecules and Fas expression on tumor cells, thereby increasing their susceptibility to cytotoxic CD8+ T cells and NK cells (31). These complementary cytotoxic pathways enable γδ T cells to contribute to cervical cancer immune surveillance even when individual recognition or death-receptor pathways are impaired.

### 4.2 Cytokine secretion and remodeling of the antitumor immune microenvironment

γδ T cells also participate in cervical cancer immune surveillance by producing a broad range of cytokines and chemokines that shape the tumor immune microenvironment. Effector γδ T cells secrete IFN-γ and TNF-α, which can promote the polarization of macrophages toward an M1-like phenotype, enhance antigen-presenting capacity, and support the establishment of Th1-dominant antitumor immunity (32–34). Through these effects, γδ T cells may help counteract the immunosuppressive milieu that commonly develops in cervical tumors. However, the cervical cancer TME can profoundly alter this cytokine-producing function. High concentrations of IL-10 and TGF-β suppress the production of IFN-γ and TNF-α by γδ T cells, thereby weakening their capacity to activate macrophages, support dendritic-cell function, and promote cytotoxic T-cell responses (35). M2-like macrophages and Tregs further reinforce this suppressive cytokine milieu through sustained production of IL-10, TGF-β, and other inhibitory mediators (36, 37). In addition, IL-6-driven STAT3 signaling may contribute to the development of dysfunctional γδ T-cell states, linking myeloid-cell-derived inflammatory signals with impaired antitumor immunity (38, 39). Metabolic conditions within the TME further shape the cytokine profile of γδ T cells. Lactate accumulation and extracellular acidification resulting from tumor glycolysis can impair IFN-γ production while favoring immunoregulatory programs, including increased IL-10 secretion (40). This shift reduces the ability of γδ T cells to sustain an inflammatory antitumor environment and may instead reinforce local immune suppression. Consequently, tumor metabolism can indirectly convert γδ T cells from immune-surveillance effectors into functionally restrained or regulatory populations.

IL-17-producing γδ T cells, which are frequently associated with Vδ1-like tissue-resident populations, may exert context-dependent functions in cervical cancer (8, 41). On the one hand, IL-17 can promote angiogenesis and facilitate the recruitment or expansion of myeloid-derived suppressor cells ( (42)s), thereby supporting tumor progression (43, 44). On the other hand, IL-17 may enhance acute inflammatory responses and neutrophil recruitment under certain conditions. Thus, the biological consequences of γδ T17 responses depend strongly on the cellular composition and signaling context of the tumor microenvironment (45). γδ T cells can also secrete GM-CSF and chemokines such as CCL5, thereby promoting the recruitment of dendritic cells and effector T cells into the tumor microenvironment and facilitating coordinated antitumor immune responses (46). Such chemokine-mediated interactions may contribute to the development or maintenance of immune-cell aggregates and, potentially, tertiary lymphoid structures. Conversely, γδ T-cell activity can itself be reinforced by cytokines derived from other innate immune populations. IL-15 and IL-18 produced within the tumor microenvironment may enhance γδ T-cell activation, proliferation, IFN-γ production, and cytotoxicity (47). These bidirectional interactions indicate that γδ T cells function as integral components of a broader immune network rather than as isolated cytotoxic effectors.

### 4.3 Antigen-presenting and helper immunoregulatory functions

In addition to their direct effector functions, γδ T cells can acquire antigen-presenting and helper-like properties, thereby linking innate and adaptive antitumor immunity. Upon activation, subsets of γδ T cells may upregulate MHC class II molecules together with the costimulatory molecules CD80 and CD86, enabling them to process and present antigens to conventional CD4^+^ αβ T cells (28, 48). Through this mechanism, γδ T cells may contribute to the initiation and amplification of tumor-specific adaptive immune responses. γδ T cells can also interact with dendritic cells through the CD40/CD40L axis, promoting dendritic-cell maturation and IL-12 production and thereby enhancing the priming and activation of cytotoxic CD8^+^ T cells (49–52). This bridging function may be particularly relevant in HPV-associated cervical cancer, in which effective coordination between innate sensing and HPV antigen-specific adaptive immunity is required for sustained tumor control. At the same time, IFN-γ produced by γδ T cells may induce the expression of immune-regulatory molecules such as PD-L1 and indoleamine 2, 3-dioxygenase (IDO) in tumor cells or other cells within the microenvironment (53, 54). Furthermore, γδ T cells can mediate antibody-dependent cellular cytotoxicity through Fc receptor-dependent mechanisms. CD16-expressing γδ T cells can recognize antibody-coated tumor cells and trigger cytotoxic responses, providing a mechanistic basis for combining γδ T-cell therapy with tumor-targeting antibodies (55, 56). In HPV-associated cervical cancer, this property may potentially complement antibody-mediated immune responses and further broaden the antitumor functions of γδ T-cell subsets (**Figure 2**).

**Figure 2:**
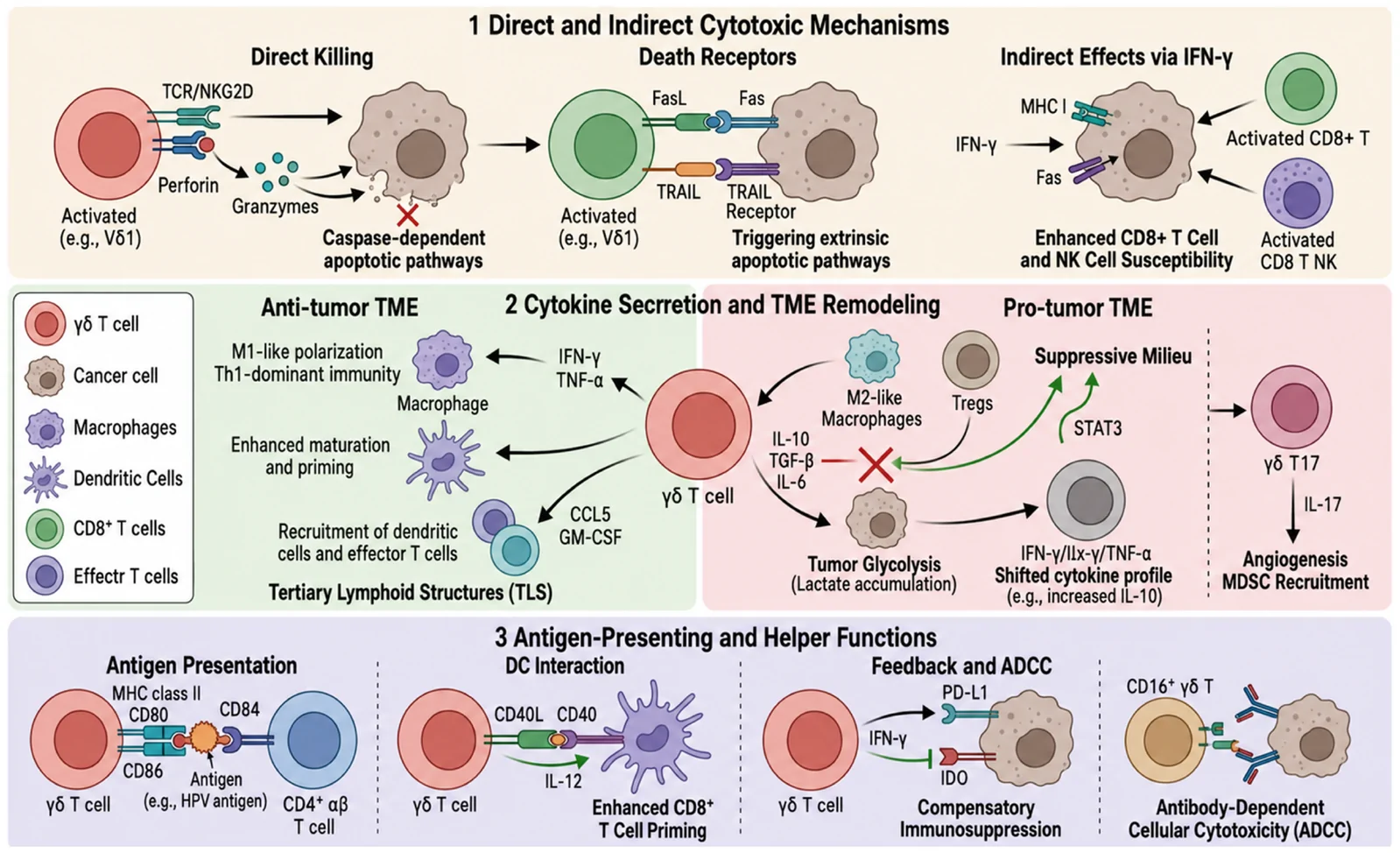
Effector mechanisms of γδ T-cell subsets in immune surveillance of cervical cancer.

## 5 γδ T cell subset-based immunotherapeutic strategies and prospects for clinical translation in cervical cancer

### 5.1 Clinical progress in ex vivo expansion and adoptive transfer of γδ T cells

Adoptive cell therapies based on γδ T cells have shown preliminary therapeutic potential in cervical cancer (50, 57). The most commonly used strategy involves the ex vivo expansion of Vδ2 T cells from peripheral blood using zoledronic acid in combination with IL-2, which can induce a 100- to 1, 000-fold increase in cell numbers (33). Several phase I/II clinical trials, including NCT02425735, have preliminarily evaluated the safety and feasibility of this approach in patients with recurrent or metastatic cancer, with reported disease control rates of approximately 30%–40% (58). However, the limited therapeutic efficacy of Vδ2 T cell-based approaches has prompted increasing interest in other γδ T-cell subsets. More recently, technical advances have enabled efficient ex vivo expansion of allogeneic Vδ1 T cells (27, 59). Stimulation with the anti-CD3 monoclonal antibody OKT3 in combination with IFNγ and TNFα can generate highly purified (>90%) functional Vδ1 T cells from healthy donor umbilical cord blood or peripheral blood, providing a foundation for the development of universal, off-the-shelf γδ T-cell products (59). Nevertheless, the optimal route of administration, cell dose, and treatment schedule remain to be established. Most current studies use repeated infusions with low-dose IL-21 administered after infusion to support cell survival and functional persistence *in vivo* (60). Short persistence after adoptive transfer together with inefficient tumor homing remains a major obstacle to therapeutic efficacy. Engineering strategies designed to enhance the expression of chemokine receptors such as CXCR4 or CCR5 may improve the directional migration and intratumoral accumulation of transferred γδ T cells (61–63).

### 5.2 CAR-γδ T cells

Chimeric antigen receptor-engineered γδ T cells combine the intrinsic MHC-independent cytotoxicity of γδ T cells with the antigen specificity conferred by CARs (64), thereby providing an attractive platform for cervical cancer immunotherapy. CAR-Vδ1 T cells targeting HER2, EGFR, and mesothelin have demonstrated substantial antitumor activity in preclinical xenograft models of cervical cancer (65). Compared with conventional CAR-αβ T cells, CAR-γδ T cells are considered to have a reduced potential for graft-versus-host disease and may exhibit a more favorable inflammatory cytokine profile, owing in part to their MHC-independent recognition and low alloreactivity. These properties make CAR-γδ T cells particularly attractive for the development of universal, allogeneic “off-the-shelf” cellular therapies (64, 66, 67). The identification of cervical cancer-specific CAR targets remains an active area of investigation. Potential targets include HPV E6/E7-derived antigens recognized through TCR-like CAR designs, MICA/MICB targeted through NKG2D-based CARs, and fibroblast activation protein (FAP) expressed within the tumor stroma (68–70). Dual-targeting CAR systems, such as those simultaneously recognizing NKG2D ligands and HER2, may further reduce the likelihood of antigen-loss-mediated tumor escape (71). In addition, CRISPR/Cas9-based gene editing has been explored to disrupt inhibitory receptors such as PD-1 and TIGIT in CAR-γδ T cells, thereby enhancing antitumor activity (72, 73). Engineering these cells to express cytokines or chemokines, including IL-15 or CCL19, may further improve their persistence, proliferative capacity, and ability to recruit endogenous immune cells within the tumor microenvironment (74–77).

### 5.3 γδ T cells-based combination immunotherapy

Because the efficacy of γδ T-cell monotherapy remains limited, combination strategies have become a major focus for improving therapeutic outcomes. PD-1/PD-L1 inhibitors, including pembrolizumab, have been approved for selected patients with cervical cancer; however, objective response rates remain modest (78, 79). Combining immune checkpoint blockade with adoptively transferred tumor-infiltrating Vδ1 T cells may enhance treatment efficacy by simultaneously restoring γδ T-cell function and increasing the number of available effector cells (1, 8, 80). Preclinical studies have suggested that anti-PD-1 therapy combined with *ex vivo*-expanded Vδ2 T cells can more effectively suppress tumor growth in HPV-positive cervical cancer models than either treatment alone (8, 56, 81). Combination therapy has also been associated with increased intratumoral CD8^+^ T-cell infiltration and higher IFN-γ levels, supporting a potential synergistic interaction between γδ T-cell therapy and immune checkpoint blockade (82). Therapeutic HPV vaccines, such as VGX-3100 and TA-CIN, are designed to induce cellular immune responses against HPV-associated antigens and may also influence γδ T-cell activation (14, 83, 84). Expansion of circulating Vδ2 T cells after vaccination has been proposed as a potential correlate of clinical response, raising the possibility that γδ T cells could serve both as therapeutic effectors and as biomarkers of vaccine-induced immunity (22). Similarly, conventional chemotherapeutic agents such as cisplatin and paclitaxel can induce immunogenic cell death, characterized by calreticulin exposure and HMGB1 release, potentially increasing tumor immunogenicity and facilitating γδ T cell-mediated immune responses (85–87). These observations provide a rationale for sequential or combinatorial chemo-immunotherapeutic strategies involving γδ T cells.

### 5.4 Microenvironment-targeted strategies to enhance γδ T-cell function

The metabolic and cytokine networks within the tumor microenvironment represent major barriers to effective γδ T-cell function. Therapeutically targeting these suppressive pathways may therefore improve the efficacy of γδ T-cell-based immunotherapy (10, 14). Inhibition of indoleamine 2, 3-dioxygenase (IDO), for example, may reduce local kynurenine accumulation and restore γδ T-cell proliferation and IFN-γ production. The IDO inhibitor epacadostat in combination with γδ T-cell transfer has been reported to suppress tumor growth in preclinical cervical cancer models (88, 89). Tumor hypoxia represents another major immunosuppressive factor. Pharmacological inhibition of HIF-1α using agents such as PX-478 may improve metabolic fitness and cytotoxic activity of γδ T cells under hypoxic conditions, although potential systemic toxicity associated with HIF inhibition must be carefully considered (90, 91). Metabolic modulators capable of restoring mitochondrial function may also enhance γδ T-cell activity. Metformin, for example, has been reported in preclinical studies to improve the survival and antitumor function of Vδ2 T cells through regulation of the AMPK–mTORC1 axis (92, 93). Cytokine-based approaches provide an additional strategy for overcoming microenvironmental suppression. Local administration of IL-15 superagonists, such as ALT-803, may preferentially expand Vδ1 T cells and enhance NKG2D expression within the tumor microenvironment. Combination with radiotherapy may further promote local tumor antigen release and generate an *in situ* vaccination effect capable of inducing systemic antitumor immunity (94, 95). Overall, these approaches aim to remodel the tumor microenvironment into a more permissive niche for γδ T-cell survival, trafficking, and effector activity.

### 5.5 Biomarkers and optimization of personalized γδ T cell-based therapeutic strategies

Accurate identification of patients most likely to benefit from γδ T-cell-based therapy will be essential for improving clinical efficacy. Candidate predictive biomarkers include the intratumoral Vδ1/Vδ2 ratio, the proportion of cytotoxic GZMB^+^ Vδ1 effector cells, and the frequency of PD-1^+^TIM-3^+^ exhausted γδ T-cell populations (96–98). However, these biomarkers require validation in prospective clinical cohorts before they can be incorporated into routine patient stratification. Elevated circulating levels of soluble NKG2D ligands, particularly soluble MICA, may also serve as indicators of impaired γδ T-cell function. Soluble MICA can interfere with NKG2D-mediated activation and promote receptor downregulation, potentially reducing the responsiveness of γδ T cells to tumor cells (99, 100). Thus, measurement of soluble NKG2D ligands may help identify patients with a highly suppressive immune environment. Single-cell transcriptomic profiling combined with TCR sequencing may enable longitudinal tracking of adoptively transferred γδ T-cell clones and provide insights into their differentiation, persistence, and functional evolution following therapy. Liquid biopsy approaches, including circulating tumor DNA analysis, may simultaneously monitor tumor burden, mutational dynamics, and the emergence of immune-escape variants (101). Furthermore, artificial intelligence-based integration of transcriptomic, proteomic, and metabolomic datasets may facilitate the molecular classification of cervical cancer and identify patient subgroups most likely to respond to γδ T-cell therapy (8). Collectively, the integration of cellular phenotyping, soluble biomarkers, single-cell multi-omics, and computational modeling may enable γδ T-cell therapy to transition from a largely uniform treatment strategy toward a more individualized and biomarker-guided therapeutic paradigm.

## 6 Conclusion

γδ T cell subsets constitute a functionally diverse component of immune surveillance in cervical cancer and occupy a unique position at the interface between innate and adaptive immunity. Unlike conventional αβ T cells, γδ T cells recognize transformed or stressed cells largely independently of classical MHC restriction, enabling rapid responses to HPV-associated epithelial transformation. Vδ1 T cells are enriched in mucosal and epithelial tissues and mainly act as tissue-resident sentinels, recognizing stress-induced ligands through receptors such as NKG2D and contributing to local tumor surveillance. In contrast, circulating Vγ9Vδ2 T cells sense phosphoantigen accumulation associated with dysregulated mevalonate metabolism and display strong cytotoxic activity through perforin–granzyme release, death-receptor signaling, and cytokine production. Vδ3 T cells are less abundant and remain incompletely characterized, but emerging evidence suggests that they may also participate in antitumor immunity. Beyond direct tumor-cell killing, γδ T cells can promote dendritic-cell maturation, enhance CD8^+^ T-cell and NK-cell responses, modulate macrophage polarization, and mediate antibody-dependent cellular cytotoxicity, highlighting their broader role as coordinators of cervical antitumor immunity.

However, the antitumor functions of γδ T cells are highly plastic and can be markedly impaired by persistent HPV infection and the immunosuppressive tumor microenvironment. HPV-derived oncogenic signals, particularly those associated with E5, E6, and E7, can alter stress-ligand expression, interfere with immune recognition, and contribute to chronic stimulation and functional exhaustion. At the same time, Tregs, MDSCs, M2-like macrophages, TGF-β, IL-10, hypoxia, lactate accumulation, and extracellular matrix barriers collectively restrict γδ T-cell trafficking, activation, cytokine production, and cytotoxicity. Under sustained suppressive conditions, γδ T cells may shift from cytotoxic effector states toward exhausted, regulatory, or IL-17-producing phenotypes, which may partially explain the inconsistent prognostic significance of γδ T-cell infiltration in cervical cancer. Future studies should therefore focus on subset-resolved functional profiling, optimization of Vδ1- and Vδ2-specific expansion, enhancement of tumor homing and persistence, and reversal of microenvironment-induced dysfunction. Integrating γδ T cell-based therapies with immune checkpoint blockade, metabolic reprogramming, cytokine modulation, or therapeutic HPV vaccination may provide a more effective strategy for achieving durable and individualized immune control of cervical cancer.
